# True Allergies to Articaine: A 25-Year Analysis

**DOI:** 10.3390/dj13050180

**Published:** 2025-04-22

**Authors:** Frank Halling, Andreas Neff, Axel Meisgeier

**Affiliations:** Department of Oral and Craniomaxillofacial Surgery, UKGM GmbH, University Hospital Marburg and Faculty of Medicine, Philipps University, 35043 Marburg, Germany; dr.halling@t-online.de (F.H.); neffa@med.uni-marburg.de (A.N.)

**Keywords:** adverse drug reaction, allergology, challenge test, local anesthesia, skin test

## Abstract

**Background**: Although allergic reactions to amide local anesthetics (LA) are rare, it is important for medical professionals to be aware of their potential occurrence. Despite the fact that articaine is one of the most commonly used LA in dentistry, a survey of documented cases of allergies to articaine is absent from the literature. The objective of this review was to ascertain how often true allergies to articaine, verified by standard allergological procedures, have been reported over the last 25 years. **Methods**: A comprehensive review of the literature from 1 January 2000 to 31 December 2024 was conducted using the PubMed-MEDLINE database. The search was limited to articles investigating suspected allergy to articaine. The search strategy encompassed indexing terms, keywords, and free-text words, complemented by an extensive manual search. The final determination was based on the application of skin and/or challenge tests as the gold standard for identifying articaine as the causative agent. **Results**: During the investigation period, 10 case reports and six case series, encompassing 29 patients with a confirmed true allergy to articaine, were identified. The age of the patients ranged from 8 to 65 years, with a median age of 34 years. Of these patients, 20 were diagnosed with an immediate type I allergy, while 5 patients exhibited a delayed type IV allergy. In four cases the specific type of allergy was not mentioned. In the majority of cases an intradermal test (IDT) was employed to ascertain the specific type of allergy. Cross-reactivity with other amide LA was reported in three cases. **Conclusions**: True allergies to articaine are exceedingly rare. Investigation strategies should include a combination of a thorough clinical history and a standardized allergological procedure entailing skin tests and challenge. Only a comprehensive approach ensures the accurate identification of affected patients and facilitates the selection of a tolerated LA.

## 1. Introduction

A safe and reliable local anesthetic is an indispensable prerequisite for contemporary dentistry and oral surgery. Local anesthetics (LA) have become one of the most prevalent medications used in daily dental in- and outpatient treatment settings, exhibiting a very low incidence of severe adverse reactions (ADRs) [1,2]. Despite the generally good tolerance to LA and their routine use, providers must remain vigilant in considering the potential for ADRs. The first analysis of the safety profiles of LA in a non-selected population, using data collected in a pharmacovigilance database, was reported by the French Association of Regional Pharmacovigilance and the GERAP database. The analysis, conducted between 1995 and 2006, identified 727 reports in which LA were suspected to be the cause of 1157 different ADRs. Lignocaine (36.0%) and bupivacaine (35.4%) were most commonly involved [3]. In a Chinese study almost half of the medical emergencies in dental clinics occurred during local anesthesia [4]. These ADRs comprised symptoms not related to LA, e.g., psychogenic reactions or dose-related toxic responses in normal individuals, and responses in susceptible patients included idiosyncratic and true allergies [5]. Several studies have demonstrated the occurrence of true allergic reactions in more or less than 1% of the patients during or shortly after administration of LA [1,6,7,8,9]. The presence of additional ingredients in LA solutions, such as antioxidants or preservatives like metabisulfite or paraben, has been demonstrated to potentially induce allergic or other adverse reactions [10]. It is imperative to acknowledge the potential challenges in distinguishing between psychogenic and allergic reactions. A Danish study indicated that the likelihood of allergic reactions to LA is frequently overestimated [11]. In certain instances, these symptoms are attributable to the properties of epinephrine, which can manifest as symptoms such as an “adrenaline rush” or vasovagal reactions [12,13,14]. The possibility of an allergic reaction, with the risk of potentially life-threatening anaphylaxis, is the most significant cause of concern and anxiety for patients with a history of an unwanted ”hypersensitivity” reaction to LA. Consequently, patients without a proper follow-up examination will be left subject to unnecessarily painful treatments or costly and time-consuming general anesthesia procedures with potential serious side-effects or even the risk of re-exposure to other unidentified allergens [15]. An allergy to LA that has not been clarified is not only relevant for the patient, but it also affects the therapeutic decisions of surgeons, anesthesiologists and dentists [7]. Allergies comprise only 2,5% to 10% of all ADRs to LA [16]. Despite the frequent use of LA, only a limited number of compelling case reports have been published on presumed IgE-mediated allergies to various LA solutions [9,17,18,19]. Allergologists have made it clear that the term “drug allergy” should be restricted to patients with a proven immunological cause [20]. According to the responsible immunological pathomechanism as well as the onset of symptoms, an immune-related drug allergy could be an immediate IgE-mediated reaction (type I) or a delayed T-cell mediated reaction (type IV) [21]. In the case of a suspected type I allergy to LA, the most useful tools to prove a true allergy are skin testing (prick and intradermal tests) as well as the provocative subcutaneous challenge test. Typically, skin-prick testing (SPT), also called a puncture or scratch test, is performed with undiluted LA and controls. For intradermal testing (IDT), preservative-free, single-dose vials are utilized. The evaluation of skin test sites is conducted after 15 min according to international guidelines and standardized protocols [22]. Subcutaneous provocation or challenge testing (SCCT) is routinely performed in skin test-negative patients as the gold standard to definitely exclude or prove an allergy [23]. For the assessment of a delayed-type reaction (type IV), patch testing with a standard of contact allergens is recommended [24,25].

Although articaine is one of the most prevalent dental anesthetics [13,26], the frequency of allergies to this drug is not known. The purpose of this study was to determine the number of cases of confirmed allergic reactions to articaine in patients within the preceding 25 years. 

## 2. Materials and Methods

A comprehensive electronic search of the English-language literature, encompassing English abstracts, was conducted using the bibliographic database PubMed-MEDLINE. The search strategy was to identify clinical studies and case reports concerning proven allergies to articaine.

The timeframe included publications from 1 January 2000 to 31 December 2024. The literature research was based on the following indexing terms, keywords, and free-text words: “allergy”, “allergic reaction”, “anaphylactic”, “local anesthetics”, “articaine”, “carticaine”, “dental”, “dentistry”, “hypersensitivity”, “intradermal test”, “LA”, “local anesthesia”, “local anesthetics”, “patch test”, “prick test”, “skin test”, “true allergy”. Additionally, the database was browsed by means of the “related articles” function and by checking the publications of authors who are well known in this scientific area. Finally, manual research was also conducted. A meticulous examination of the listed references of the selected articles was conducted to identify additional relevant publications. The aim of our study was to create an overview of all cases of dermatologically approved allergies to articaine within the timeframe of 25 years. The crucial inclusion criterion was the detailed documentation of an allergy by at least one positive result from skin tests, challenge tests or patch tests. Additional information like clinical history, the kind of tests performed, clinical symptoms and reactions and proven cross-reactivity was also taken into consideration during the analysis.

## 3. Results

During the 25-year investigation period, only 10 case reports and six case series were identified, encompassing 29 patients with documented authentic allergies to articaine (Table 1 and Table 2).

In eighteen cases, the patient’s age and gender were documented. These cases included seven men and eleven women, reflecting a gender distribution of 1.57 (♀): 1 (♂). The ages of the patients ranged from 8 to 65 years, with a mean age of 33.9 years (SD 17.3). On average the women affected were 7.4 years older than the men (♀ 34 y vs. ♂ 26.6 y). However, in eleven cases, pertinent information regarding age and gender was not documented. It is noteworthy that all patients with reported clinical histories had previously undergone at least one dental treatment that involved the application of articaine for local anesthesia. The onset of suspected allergy reactions occurred either within one hour or after more than 24 h. In all cases allergies were definitely confirmed through various types of positive skin tests, subcutaneous challenge tests or patch tests. Twenty patients were diagnosed with an IgE-mediated type I allergy and five patients were reported to have a delayed type IV allergy, including one patient with a fixed drug eruption. However, in four cases the specific type of allergy was not specified. SPTs were performed in three cases, IDTs in eleven cases and SCCTs in nine cases. In order to verify a delayed type IV allergy, a patch test was conducted in three cases. In ten patients a type I allergy was confirmed solely by IDT, while SCCTs were used exclusively in six patients and in combination with a SPT in two patients. The patch tests yielded two positive results; however, in the case of a fixed drug eruption, only the SCCT was positive, while the patch test was negative. In one case report and one retrospective analysis involving five patients, the specific kind of skin testing was not specified, and the results were only reported as “positive”. The spectrum of clinical signs associated with skin testing was very broad and ranged from swelling, discomfort and pruritus to angioedema and generalized urticaria and edema. The duration of allergic symptoms did not exceed 15 days. Notably, anaphylactic reactions or death were not observed in any of the cases. Cross-reactivity among the amide family occurred in three cases. However, with the exception of one case, other amides were tolerated [23].

## 4. Discussion

Despite the fact that articaine is one of the most frequently administered local anesthetic agents in dentistry worldwide [13,34], with market shares of up to 98% [13], there is little information on the type and frequency of allergies to this drug. In contrast to lignocaine, which came on the market in the late 1940s, articaine was developed nearly 30 years later in Germany and is considered a better tolerated and more efficient alternative to lignocaine [36,37,38,39,40,41]. A unique property of articaine is its rapid metabolization to an inactive form in both plasma and tissues, due to the presence of the hydrolyzable ester group [37,41].

After the release of articaine, the first case reports of allergies to it were published in the late 1980s and early 1990s [42,43]. It quickly became apparent that articaine, like other amide-type local anesthetics, is less allergenic than ester-type anesthetics [44]. In the six case series analyzed in our review, allergies to lignocaine occurred about twice as often as allergies to articaine (40 vs. 19 cases) [3,7,23,25,34,35]. Because the guidelines for standardized reproducible skin testing with safe and nonirritant drug concentrations were only published in 2013 [22], most of the case series concerning allergies to LA were released from that year onwards [7,23,25,35]. But until now a compilation specifically addressing allergies to articaine has been missing.

In all case reports in our review the patients were referred by a dentist. The most common reason was a somatic reaction of the patient during or after dental treatment after administration of articaine. The spectrum of clinical symptoms was very diverse, ranging from mild symptoms like skin erythema to severe reactions such as angioedema or hypotension and tachycardia. For a thorough evaluation, the history of the reported reaction; the onset, type and duration of the symptoms; the drugs used; and the temporal course of the event are very helpful in diagnosing a true allergic reaction [44]. Urticaria, eczema, angioedema, bronchospasm and cardiovascular depression are the main symptoms of immediate type I reactions [45]. No cases of life-threatening anaphylactic reactions or death were reported in our patient group. However, urticaria or eczema, swelling and angioedema may also be triggered by a delayed T-cell-mediated immune response [24,25,35]. Therefore, differentiating between type I and the rare type IV reactions exclusively through clinical symptoms is not possible. We found a distinct propensity among female subjects to develop allergies to articaine. The reason for this noticeable finding is unclear [3,34]. Like in existing case series reports, the age range of our group of patients is relatively broad [3,34].

ADRs commonly have another non-allergic mechanism, such as central nervous reflex reactions, or are caused by simultaneous exposure to other materials such as chlorhexidine or latex. The fact that psychological stress and fear of the procedure itself may lead to psychogenic symptoms and can contribute to adverse general reactions should not be neglected [11,15,21]. Psychogenic reactions frequently mimic allergic reactions because of similar symptoms like tachycardia and hypotension as well as concomitant nausea, dizziness, sweating or hyperventilation [1]. Consequently, it is not feasible to definitely confirm a diagnosis of “true allergic reaction” solely on the basis of anamnesis and reported symptoms. Positive skin tests are mandatory as additional diagnostic modalities [3].

It is imperative to note that local anesthetics may contain known allergens such as the bacteriostatic methylparaben and the antioxidant metabisulfite. Currently, the utilization of methylparabens in dental cartridges has been discontinued in many countries, as they are single-patient-use medications. In some cases, testing with both paraben-free and paraben-containing LA may be appropriate [46]. However, metabisulfite, an added antioxidant, remains in all solutions containing epinephrine [47]. Allergic responses to metabisulfite in anesthetic solution are rare [48,49]. In our study, epinephrine or metabisulfites were only tested as single active ingredients or vasoconstrictor-free LA solutions in order to determine hypersensitivity to epinephrine or antioxidants in some cases [19,23,24,28,35]. Aksu and Kurt mentioned that no product containing articaine without epinephrine is available in the Turkish market [19]. Local anesthetics devoid of vasopressors (e.g., mepivacaine 3%) do not contain sulfites, thereby eliminating the potential for sulfite-based allergic reactions. In patients with food allergies associated with sulfites, local anesthetics containing vasoconstrictors should be used cautiously or withheld, given the potential for severe reactions [48]. Total sulfite-based allergic reactions are encountered in 2% of the general population, with 4–10% occurring in patients with asthma, and they can occur in 20–40% of the patients who do not tolerate aspirin [50].

In rare cases of skin reactions after application of LA, mast cell involvement must be considered as a causal mechanism [15,23]. Mastocytosis is a disease characterized by the proliferation and accumulation of mast cells in various tissues, preferentially skin and bone marrow, leading to a wide variety of clinical manifestations, and is mainly caused by the inappropriate release of mast cell mediators. As drugs are possible triggers of anaphylaxis in patients with mastocytosis, mast cell disorders may be excluded in cases of severe systemic reactions to drugs. The most frequent triggers of anaphylaxis in the context of mast cell disorders are food (29%), insect stings (22%), and drugs (15%), but published data on drug anaphylaxis in patients with mast cell disorders are scarce. Currently, it is not possible to provide clear recommendations [51].

According to the literature, SPT and IDT were the main diagnostic methods used in our patient group to identify the causative agent in patients with suspected perioperative anaphylaxis [5,8,22,23]. Currently, IgE-mediated LA allergies can neither be diagnosed nor excluded by laboratory testing [23]. Therefore, SPT is generally performed first, as this carries a very low risk of ADRs, and is subsequently followed by IDT, which is associated with a slightly higher risk of serious ADRs. In our study, IDT was used to verify the allergy in most cases of IgE-mediated type I allergy. There were no cases where SPT was used as the sole testing procedure. The diagnostic value of both testing procedures was compared in a clinical study with 212 patients [52]. The authors found an overall agreement of 93% between the paired tests. The differences between the tests were not statistically significant. The authors concluded that skin testing is a valuable tool in the evaluation of allergies associated with anesthetics, but when in doubt, both tests should be performed [52]. However, skin tests, especially IDT, are painful and less well tolerated by children. They provide a low sensitivity level in mild nonimmediate skin reactions and the true rates of false positives and negatives remain unclear [23,45,53]. Therefore, drug provocation tests such as SCCT with allergen re-exposure are often considered to be the gold standard or the final step in confirming or excluding a drug allergy or in finding safe alternatives in case of confirmed hypersensitivity to the culprit drug [9]. SCCT requires the possibility of the timely and efficient initiation of rescue and security measures, along with effective on-site emergency medical care. Some authors suggest provocation tests without previous skin testing in an inpatient setting under the acute supervision of an experienced clinician [54]. It is important to use only one specific local anesthetic for the drug provocation test, as this minimizes the risk of anaphylactic reactions [9,23,53,55]. In our study, SCCT was performed without a previous SPT or IDT in nine cases. Provocation testing has been reported to have a very good negative predictive value in both children and adults [53,55].

According to Koca Kalkan et al., a reaction is classified as a delayed- or late-type reaction if its symptoms occur > 6 h after provocation [8]. It is supposed that LA very rarely trigger delayed systemic symptoms like urticaria or eczema [35]. An IDT may be used to diagnose a delayed-type immune response if readings are taken after 1–2 days [35,45]. Also, patch tests are simple, cheap and effective diagnostic tests for investigating delayed hypersensitivity reactions to LA [24,45]. In our retrospective analysis we found five cases with a type IV allergy. Each patch test was performed by administering one or two drops of undiluted solution to patch test chambers fixed on the back skin and readings were taken after 48 and 72 h [24].

In two cases the patch tests were positive after 48 h [24,25]. In one case only the IDT was positive after 24 h; in another, which had a false negative skin result, only the SCCT was positive [35]. In one case report, the authors were able to document a fixed drug eruption by a positive SCCT and histology [32]. To date, this is the first and only documented fixed drug eruption provoked by articaine.

The ENDA/EAAC guidelines strongly recommend that in cases of confirmed LA allergies, other LA should be tested to identify an alternative [22]. Due to different breakdown products, cross-reactivity is less frequent between amide group LA and ester group LA [8,45]. Cross-reactivity among amide LA has been reported, but a specific pattern has not been identified [8,23,24,31,35,55,56]. Because of the different metabolites within the amide group, it is supposed that cross-reactivity may be due to different sensitizations with more than one allergen structure [8]. We found only three reports of cross-reactivity between articaine and other amides, but in two cases alternative amide-type LA were tolerated.

The main limitation of our study is the heterogeneity of data from different sources and the retrospective design. To date, only a single prospective study has been published that evaluates the incidence of immediate adverse events in subjects requiring local anesthetic injections. Despite the substantial number of participants, totaling more than 5.000, none of the observed 25 reactions were attributable to an allergic cause [57]. In the present study, the test procedures employed to verify allergies did not consistently adhere to recommended guidelines, especially concerning the heterogeneity of concentrations used for intradermal testing, the different reading times, the tests for cross-reactivity and the exclusion of other potential allergens like preservative agents or latex. In this context, Campell et al. point out that the most probable causes of an allergic response are the preservative, antioxidant or metabolites and not the anesthetic itself [48]. Another major limitation is the voluntary reporting system, which can lead to the under-reporting of ADRs. A recent systematic review revealed that low ADR reporting is primarily associated with a series of attitudes (ignorance, lethargy or complacency) and excuses [58]. Notably, even in countries with a specific legal obligation to report ADRs, like France, the rate of reported serious ADRs is estimated to be a mere 5% [59]. An Indian study revealed that oral health practitioners have a general understanding of ADRs, but there is substantial evidence of underreporting and a lack of reporting system information [60].

The objective of this review was to ascertain how often true allergies to articaine, verified by standard allergological procedures, have been reported over the last 25 years. The final number of cases, 29, is very small relative to the massive usage of articaine throughout the world. Nevertheless, it is of particular importance that potential reactions to LA procedures are not hastily labeled as allergic. But if an allergy to articaine is suspected, it is up to the dentist to refer the patient to an allergy specialist or specialist clinic. Standardized allergological procedures, i.e., SPT or IDT for all drugs the patient was exposed to, should be performed. In cases of negative skin tests, SCCT is the ultimate procedure to confirm or exclude an allergy. Also, alternative agents should not be used without thorough allergological investigation. This approach may facilitate the confirmation of the culprit agent and the identification of a suitable alternative agent. Finally, it is imperative to ensure that the patient concerned is informed about any LA that should definitely be avoided and those that are permissible for further treatment.

## Figures and Tables

**Table 1:** The main characteristics of the case reports and case series included in the present review.

Authors (Alphabetical)	Year of Publication	No. of Patients with Allergy to Articaine/No. of Patients in the Study	Patient Selection—Clinical History
**Aksu and Kurt** [19]	2012	1/1 (case report)	Referral for allergological evaluation—quick skin and neurological reactions after application of articaine with epinephrine for dental treatment
**Alonso et al.** [27]	2022	1/1 (case report)	Referral for allergological evaluation—skin reactions within a few hours after application of articaine with epinephrine for an endodontic procedure
**Davila-Fernandez et al.** [28]	2012	1/1 (case report)	Referral for allergological evaluation—skin reactions within a few hours after application of articaine with epinephrine for an endodontic procedure
**De Pasquale et al.** [24]	2018	1/1 (case report)	Referral for allergological evaluation—skin reaction (approx. 20 min) after application of articaine with epinephrine for a dental procedure
**El-Quotob et al.** [29]	2005	1/1 (case report)	Referral for allergological examination—skin reaction after application of articaine with epinephrine in the context of a dental procedure
**Fuzier et al.** [3]	2009	2/286	Retrospective analysis of the French pharmaco-vigilance database and the GERAP database
**He et al.** [30]	2015	1/1 (case report)	Different somatic reactions 5 min after local infiltration of articaine for a dental procedure
**Kamchaisatian et al.** [31]	2014	1/1 (case report)	Referral to clinic—immediate physical reactions after application of articaine
**Kleinhans et al.** [32]	2004	1/1 (case report)	Referral to dermatology department after several episodes of delayed skin reactions after application of articaine (48 h later)
**Mehta and Barisciano** [33]	2020	1/1 (case report)	Referral to medical department for allergological evaluation—suspected allergy to articaine
**Moreno-Escobosa et al.** [26]	2011	1/1 (case report)	Referral of a patient with alleged allergic reaction to LA after 30 mins (dental procedures)
**Sambrook et al.** [34]	2011	4/227	Retrospective analysis of a database of the Australian government (1973–2008)
**Specjalski et al.** [7]	2013	6/154	Retrospective analysis of patients with a history of adverse reactions to LA (2006–7/2012)
**Trautmann et al.** [23]	2018	2/402	Retrospective analysis of referred patients with alleged allergic reactions to LA
**Trautmann and Stoevesand** [35]	2018	2/202	Referral to clinic for allergological examination of suspected LA-associated late-type reactions
**Tupker et al.** [25]	2014	3/53	Retrospective analysis of patients referred to two hospitals to clarify a suspected allergy to local anesthetics

**Table 2:** The characteristics of the documented cases of true allergies to articaine (2000–2024).

Authors (Alphabetical)	No. of Patients	Gender	Age	Kind of Test			Clinical Symptoms and Reactions	Tolerated Anesthetic	Cross Reactivity	Kind of Allergy
				SPT	IDT	SCCT				
**Aksu and Kurt** [19]	1	f	50	-	+	ND	Skin redness, globus hystericus, paresthesia in tongue	Prilocaine	-	Type I
**Alonso et al.** [27]	1	f	18	+	+	ND	Swelling and erythema, disseminated papules of thorax, abdomen and arms; angioedema of tongue and pharynx within 48 h	Lidocaine	-	Type I
**Davila-Fernandez et al.** [28]	1	f	26	-	+	ND	Generalized urticaria and dysphagia	Lidocaine, mepivacaine, bupivacaine	-	Type I
**De Pasquale et al.** [24]	1	f	42	ND	ND	ND	Eczematous reaction of the left cheek and the upper neck (lasted for 15 d)	Mepivacaine	Lidocaine, bupivacaine	Type IV
				Patch test + (after 48 h)						
**El-Quotob et al.** [29]	1	f	51	+	ND	ND	Erythema, edema of lips, face and eyelid	Lidocaine, mepivacaine, bupivacaine	-	Type I
**Fuzier et al.** [3]	2	f	22	ND	+	ND	Discomfort, pruritus	NM	NM	Type I
		f	44	ND	+	ND	Eczema, edema	Lidocaine, mepivacaine	-	Type I
**He et al.** [30]	1	m	65	“Positive skin test” without specification			Discomfort, chest distress, weak breathing, palpitation, dizziness	NM	NM	Type I
**Kamchaisatian et al.** [31]	1	m	11	ND	+	ND	Lip swelling and itching	Lidocaine, bupivacaine	Mepivacaine	Type I
**Kleinhans et al.** [32]	1	f	45	-	-	+	Dark bluish macules and plaques on both hands (histological verification of a fixed drug eruption) 48 h after testing	Lidocaine, mepivacaine	-	FDE/type IV
				Patch test -						
**Mehta and Barisciano** [33]	1	f	8	+	+	ND	Oral angioedema	NM	NM	Type I
**Moreno-Escobosa et al.** [26]	1	f	25	-	-	+	Generalized pruritus, facial edema, hives on the face, neck and thorax	Mepivacaine, lidocaine, bupivacaine	-	Type I
**Sambrook et al.** [34]	4	NM	NM	“Positive skin test” without specification			NM	NM	NM	NM
**Specjalski et al.** [7]	2	NM	NM	-	+	ND	NM	NM	NM	Type I
	4			-	-	+				Type I
**Trautmann et al.** [23]	2	m	28	ND	+	ND	Urticaria within 30 mins; hypotension, tachycardia	Mepivacaine, prilocaine, procaine	-	Type I
		m	34	ND	+	ND		Procaine	Mepivacaine, prilocaine	Type I
**Trautmann and Stoevesand** [35]	2	m	55	ND	+	ND	Redness and swelling	Prilocaine	-	Type IV
				(after 24 h)						
		m	55	ND	-	+		Prilocaine, mepivacaine	-	Type IV
**Tupker et al.** [25]	3	m	18	ND	-	+	Local redness, itching	NM	NM	Type I
		m	12	ND	-	+	Local urticaria, itching			Type I
		NM	NM	Patch test +			Generalized urticaria (face and body)			Type IV

+: positive test reaction, -: negative test reaction, NM: not mentioned, ND: not done, m: male, f: female, SPT: skin prick test, IDT: intradermal test, SCCT: subcutaneous provocation test, FDE: fixed drug eruption.

## Data Availability

The data presented in this study are available on request from the corresponding author.
